# Environmental performance of olive stone–plastic waste activated carbon composites

**DOI:** 10.1039/d6ra00017g

**Published:** 2026-02-20

**Authors:** Junaid Saleem, Zubair Khalid Baig Moghal, Furqan Tahir, Gordon McKay

**Affiliations:** a Division of Sustainable Development, College of Science and Engineering, Hamad Bin Khalifa University, Qatar Foundation Doha Qatar jsaleem@hbku.edu.qa zubairkhalid009@gmail.com

## Abstract

The circular valorization of agricultural residues and plastic waste into functional materials is a critical challenge for sustainable water treatment technologies. In this study, activated carbon (AC) composites were produced by integrating olive stone (OS) and mixed plastic waste (MPW) into a carbon–polymer adsorbent, and their environmental sustainability was systematically evaluated using life cycle assessment (LCA). The OS–MPW AC composites exhibit low environmental burdens, with climate change (CC) impacts of 3.70 kg CO_2_ eq. and an energy net (EN) of 100 MJ kg^−1^. In contrast, commercial AC shows substantially higher impacts, with CC emissions of 4.8 kg CO_2_ eq. and an EN of 125 MJ. In addition, the waste-derived composites demonstrate high adsorption performance toward Rhodamine-6G, achieving a maximum adsorption capacity of 669 mg g^−1^, thereby reducing the amount of adsorbent required for effective pollutant removal. To capture both production efficiency and application relevance, LCA was conducted using two functional units: per kg of AC composite produced and per kg of dye adsorbed. Contribution analysis identifies pyrolysis and polymer dissolution as the dominant contributors to CC and EN. Sensitivity analyses further indicate that pyrolysis energy consumption is the most influential uncertainty parameter. Overall, the results demonstrate that OS and MPW can be effectively co-valorized into high-performance AC composites with favorable environmental profiles.

## Abstract 2

The circular valorization of agricultural residues and plastic waste into functional materials is a critical challenge for sustainable water treatment technologies.

## Graphical abstract

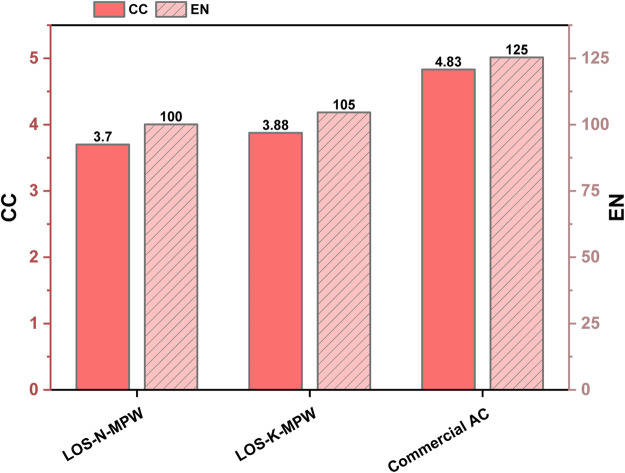

## 1. Introduction

Activated carbon (AC) is widely used in water purification, air treatment, and industrial separation processes due to its high surface area, tunable porosity, and strong affinity for organic and inorganic contaminants.^1,2^ Conventional AC production relies predominantly on fossil-based precursors such as coal, petroleum coke, and lignite, which are associated with high carbon intensity, resource depletion, and long-term environmental burdens.^3–5^ In response to these concerns, biomass-derived AC has emerged as a sustainable alternative.^6–9^

Among agricultural residues, olive stones (OS)—a byproduct of olive oil production—have attracted increasing attention as a promising precursor for AC synthesis. OS account for approximately 10–30 wt% of the olive fruit and exhibit high lignocellulosic content, low ash fraction, and relatively high fixed carbon content, making them well suited for thermochemical conversion into porous carbon materials.^10–15^ Numerous studies have demonstrated that OS-derived AC can achieve high surface areas and excellent adsorption capacities through optimized pyrolysis and chemical activation conditions.^16,17^ However, most of these studies have primarily focused on material development and adsorption performance, with comparatively limited attention paid to the environmental sustainability of the production routes and the product.

In parallel, mixed plastic waste (MPW)—particularly polyolefins such as polyethylene (PE) and polypropylene (PP)—poses a significant environmental challenge owing to low recycling rates and high chemical inertness.^18–21^ Consequently, integrating plastic waste into AC synthesis has been widely explored as a dual-valorization strategy that addresses plastic disposal while improving the physical form and handling characteristics of biomass-derived AC. Most reported studies on plastic waste valorization follow a carbonization-centric pathway, wherein plastics are subjected to high-temperature pyrolysis followed by chemical or physical activation to generate porous AC with high specific surface area for adsorption and energy-related applications.^22–29^ While these approaches are effective in producing high-surface-area carbons, they fundamentally rely on the thermal destruction of the polymer backbone.

On an alternative front, solvent-dissolution-assisted strategies provide a distinct, non-carbonization route that preserves polymer structure.^30–37^ In this approach, semi-crystalline polymers are dissolved in suitable solvents at temperatures below the solvent boiling point. Depending on the targeted application, biowastes-based AC may optionally be incorporated into the polymer solution and homogeneously dispersed. Subsequent cooling induces composite solidification and solvent separation, forming free-standing structures, while mild post-heating is applied to enhance mechanical integrity.^38–40^ These dissolution-based routes produce mechanically robust composite or flake-like morphologies that, unlike powdered AC, can be readily recovered from aqueous systems, improving their practical applicability in water treatment applications.

In parallel, advanced adsorbents and membrane-based systems—particularly those based on layered double hydroxides (LDHs) and hybrid nanocomposites—have shown strong potential for the removal of trace contaminants, including heavy metals, boron, and pharmaceutical compounds from complex water matrices.^41–43^ While such systems offer high selectivity and tunable separation performance, they typically involve multistep synthesis routes, specialized materials, and limited consideration of life cycle environmental impacts. In contrast, waste-derived AC–polymer composites provide a simpler and more scalable pathway that directly integrates adsorption functionality with circular waste valorization, motivating the present LCA-driven evaluation.

Despite the growing interest in OS-derived AC and the increasing incorporation of plastic waste into AC synthesis, no study has systematically quantified the life cycle assessment (LCA) of co-valorizing OS–MPW-derived AC composites using experimental data, leaving a critical knowledge gap in assessing the sustainability of these composite systems. Moreover, existing LCA studies on AC predominantly adopt mass-based functional units (*e.g.*, per kg of AC produced), which fail to account for variations in adsorption performance arising from differences in surface chemistry, porosity, and composite structure.

Because adsorption capacity directly governs the quantity of adsorbent required to achieve a given level of pollutant removal, performance-based functional units provide a more application-relevant and environmentally meaningful basis for comparison. The absence of such performance-normalized assessments limits the ability to make robust comparisons between conventional powdered AC and composite or structured adsorbents derived from waste materials. A combined evaluation framework integrating both mass and performance-based metrics is therefore essential to accurately assess environmental trade-offs and identify truly sustainable adsorption materials.

Accordingly, the present study conducts an LCA of AC composites produced from OS and MPW *via* chemical activation. EIs are quantified using two functional metrics: per kg of AC composite produced and per kg of dye adsorbed. By explicitly linking environmental burdens to both material production and functional performance, this work provides the LCA-driven evaluation of OS–MPW-derived AC composites, advancing environmentally informed design strategies for the circular valorization of agricultural and plastic wastes in water treatment applications.

## 2. Materials and methods

Rhodamine 6G dye, sodium hydroxide (NaOH), and potassium hydroxide (KOH) were obtained from Sigma-Aldrich (USA), while OS were collected from local sources. Initially, 1 kg of OS biomass was dried, and shredded, during which an estimated 10% mass loss occurred. The dried biomass was chemically activated using either KOH or NaOH, by immersing it in 2 M KOH or 2 M NaOH solutions overnight. The impregnated biomass was then oven-dried and subjected to pyrolysis in a tubular furnace at 550 °C with a residence time of 3 h and a controlled heating rate of 20 °C min^−1^ under a nitrogen atmosphere, resulting in 29% biochar. Nitrogen was used to maintain an inert atmosphere during pyrolysis in order to prevent biomass oxidation, maximize carbon yield, and minimize ash formation.

40 g of mixed plastic waste (MPW), consisting of polyethylene (PE) and polypropylene (PP) in an equal ratio, was dissolved in 2 L of xylene by heating the solvent to 120–130 °C, *i.e.*, below its boiling point, under continuous stirring and reflux condensation in a nitrogen atmosphere. Complete polymer dissolution was achieved within approximately 20 min, resulting in a homogeneous polymer solution. The as-produced activated char was then added and dispersed under continuous stirring until a uniform suspension was obtained. The mixture was subsequently transferred to molds and allowed to cool, leading to polymer solidification.

During cooling, solvent recovery was carried out in two stages. After composite solidification, the bulk xylene was recovered by decantation, followed by vacuum condensation to recover the remaining solvent. The recovered xylene was reused for up to three subsequent preparation cycles under laboratory conditions. After the third cycle, minor residue accumulation was observed in the round-bottom flask, and further reuse was not continued in this study.

The resulting fragile composite was finally subjected to thermal treatment at approximately 170 °C, corresponding to the melting temperature of the polymer, to enhance mechanical integrity and obtain the final carbon–polymer composite (AC composite). Regeneration of the composite after dye adsorption was performed by stirring the dye-loaded composite in 0.1 M NaOH solution overnight, followed by filtration and oven drying prior to reuse.

LCA was conducted by normalizing all material and energy flows to 1 kg of carbon–polymer composite, which was selected as the functional unit. The inventory data required to produce 1 kg of AC composite, including all input and output flows, are summarized in Table 1. The system boundary encompassed transport, shredding, oven drying, chemical activation, pyrolysis, neutralization, dissolution, recovery, and heat treatment with electricity, activating agents, plastic dissolving solvent, water, and nitrogen (for inert atmosphere) included as process inputs. After pyrolysis, residual alkaline species were neutralized using a mild 0.1 M HCl solution to rapidly remove basic residues as soluble salts, thereby minimizing water consumption compared to water-only washing. Water use and wastewater generation associated with neutralization and washing were included in the life cycle inventory and modeled using standard wastewater treatment processes available in the LCA database. Nitrogen's contribution to overall EIs is minor compared to energy-intensive thermal processing and is therefore included only as a secondary input in the life cycle inventory.

**Table 1:** Process steps and resources for AC composite production

Process	Resource allocation	Units	Activation	
			2 M KOH	2 M NaOH
**Inputs**				
Collection	Raw OS	kg	3.678	
Transport	US: transport, combination truck, gasoline powered	km	36.78	
Shredding	Shredder	MJ	0.066	
Drying-1	Hot air oven	MJ	0.530	
Activation	KOH/NaOH	g	371.45	264.8
	Water as a solvent, desalinated, deionized	kg	3.134	3.187
Drying-2	Hot air oven	MJ	0.477	
Pyrolysis	Tubular furnace	MJ	15.790	
	N_2_	kg	0.048	
Neutralization	Water as a solvent, desalinated, deionized	kg	216.96	
	HCl	g	5.745	
Drying-3	Hot air oven	MJ	0.138	
Dissolution	Xylene as a solvent to dissolve plastic	g	579.96	
	Mixed plastic waste (PE + PP)	g	40	
	Hot plate-magnetic stirrer	MJ	0.972	
Heat treatment	Oven	MJ	0.60	

**Outputs**				
Product	Activated carbon	kg	1	1
Solid and gases	H_2_O, CO_2_, CO, CH_4_, NH_3_, K_2_CO_3_, NH_3_, H_2_, ash, xylene, *etc.*	kg	3.887	3.780

**Fig. 1:**
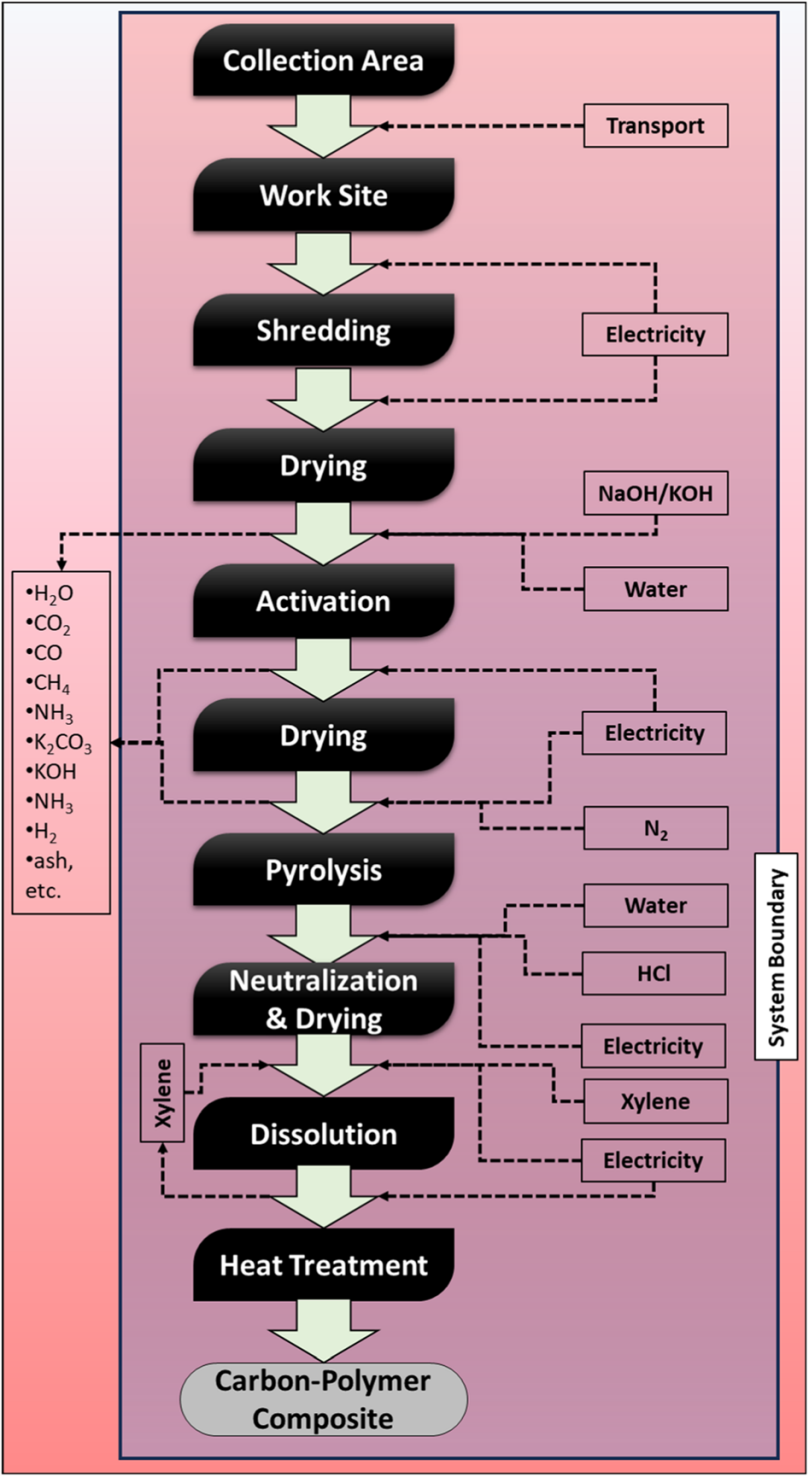
System boundary of the processes involved in the preparation of AC composite.

Emissions generated during the pyrolysis step—such as CO, H_2_, CO_2_, CH_4_, and K_2_CO_3_—were excluded from the system boundary. This exclusion was made because certain gaseous by-products (*e.g.*, CO, H_2_, and CH_4_) can potentially be recovered and reused as fuel. Consequently, these assumptions allow the analysis to focus specifically on the EIs associated with the activation and composite preparation processes (Fig. 1).

## 3. Results and discussion

The results are initially analyzed using a mass-based functional unit (per kg of AC produced) to quantify the EIs associated with material synthesis and overall process efficiency. This framework enables a direct, production-level comparison between the NaOH- and KOH-based activation routes. The assessment is then expanded to a performance-based functional unit (per kg of dye adsorbed), which incorporates variations in adsorption capacity and better represents the practical application of AC in pollutant removal.

**Fig. 2:**
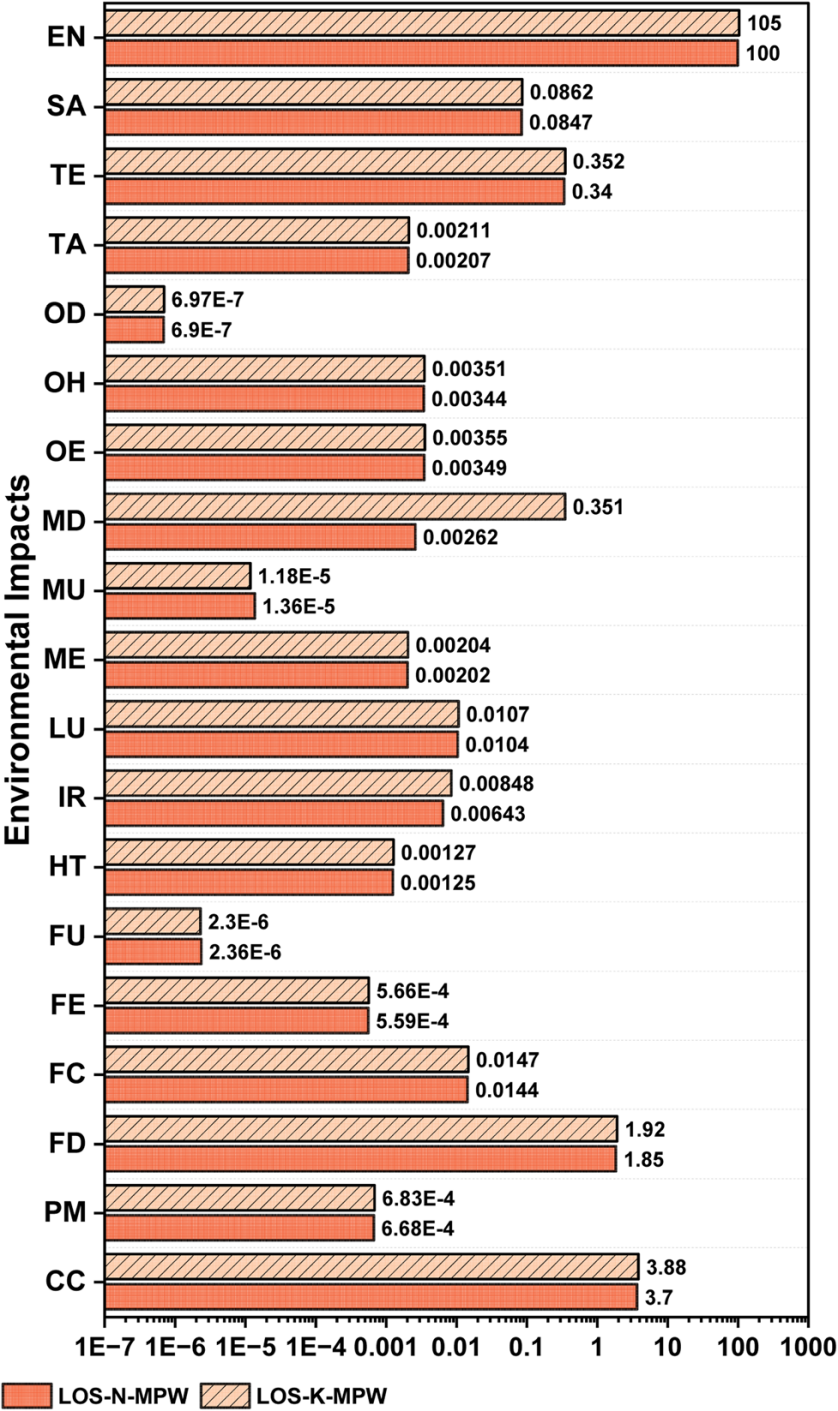
EIs during the preparation of AC composite.

Chemical activation enhances the development of porosity by promoting dehydration, cleavage of carbon–oxygen bonds, and facilitates in the removal of volatile matter during subsequent pyrolysis.^16^Table 2 and Fig. 2 compare the environmental indicators (EIs) associated with the chemical activation stage for OS–MPW AC composites produced *via* NaOH (LOS-N-MPW) and KOH (LOS-K-MPW) activation routes. Here, LOS-N-MPW denotes the LCA of AC composites using NaOH activation, while LOS-K-MPW represents the corresponding LCA using KOH activation. Among these, the most significant EIs are CC and EN. The higher carbon emissions, metal depletion, and energy demand associated with KOH stem from its production process. KOH is mainly generated through the electrolysis of potassium chloride (KCl), which requires considerably more energy than the production of NaOH. This is due to KOH's strong tendency to absorb carbon dioxide and water, necessitating a more energy-intensive procedure. Electrolyzing KCl produces KOH along with chlorine and hydrogen, consuming substantially more electricity compared to NaOH production, where membrane electrolysis of sodium chloride (NaCl) efficiently separates NaOH and hydrogen from chlorine while minimizing hazardous by-products. Furthermore, KOH is costlier because KCl is less abundant and more expensive than NaCl.

**Table 2:** Selected EI categories for the activation process

Impact category	Unit	Abbreviation	LOS-N-MPW	LOS-K-MPW
Climate change	kg CO_2_ eq.	CC	3.70 × 10	3.88 × 10
Particulate matter	kg PM2.5 eq.	PM	6.68 × 10^−4^	6.83 × 10^−4^
Fossil depletion	kg oil eq.	FD	1.85 × 10	1.92 × 10
Freshwater consumption	m^3^	FC	1.44 × 10^−2^	1.47 × 10^−2^
Freshwater ecotoxicity	kg 1,4 DB eq.	FE	5.59 × 10^−4^	5.66 × 10^−4^
Freshwater eutrophication	kg P eq.	FU	2.36 × 10^−6^	2.30 × 10^−6^
Human toxicity	kg 1,4-DB eq.	HT	1.25 × 10^−3^	1.27 × 10^−3^
Ionizing radiation	kBq Co-60 eq. to air	IR	6.43 × 10^−3^	8.48 × 10^−3^
Land use	Annual crop eq. year	LU	1.04 × 10^−2^	1.07 × 10^−2^
Marine ecotoxicity	kg 1,4-DB eq.	ME	2.02 × 10^−3^	2.04 × 10^−3^
Marine eutrophication	kg N eq.	MU	1.36 × 10^−5^	1.18 × 10^−5^
Metal depletion	kg Cu eq.	MD	2.62 × 10^−3^	3.51 × 10^−1^
Ozone ecosystems	kg NOx eq.	OE	3.49 × 10^−3^	3.55 × 10^−3^
Ozone human health	kg NOx eq.	OH	3.44 × 10^−3^	3.51 × 10^−3^
Ozone depletion	kg CFC-11 eq.	OD	6.90 × 10^−7^	6.97 × 10^−7^
Terrestrial acidification	kg SO_2_ eq.	TA	2.07 × 10^−3^	2.11 × 10^−3^
Terrestrial ecotoxicity	kg 1,4-DB eq.	TE	3.40 × 10^−1^	3.52 × 10^−1^
Smog air	kg O_3_ eq.	SA	8.47 × 10^−2^	8.62 × 10^−2^
Energy net	MJ	EN	1.00 × 10^2^	1.05 × 10^2^

Across most impact categories, the NaOH route exhibits slightly lower environmental burdens than the KOH route. In terms of climate change (CC), LOS-N-MPW shows an impact of 3.70 kg CO_2_ eq., compared to 3.88 kg CO_2_ eq. for LOS-K-MPW, corresponding to an increase of approximately 5% for KOH activation. A similar trend is observed for net energy demand (EN), which increases from 100 MJ for NaOH to 105 MJ for KOH. These differences can be attributed primarily to the higher embodied energy and upstream production impacts of KOH, as well as its higher consumption during activation and subsequent neutralization and washing steps.

Energy- and resource-related impact categories further reinforce this trend. Fossil depletion (FD) increases from 1.85 kg oil eq. for NaOH to 1.92 kg oil eq. for KOH, while freshwater consumption (FC) rises slightly from 1.44 × 10^−2^ m^3^ to 1.47 × 10^−2^ m^3^. Human toxicity (HT) and freshwater ecotoxicity (FE) also show marginally higher values for the KOH route, with HT increasing from 1.25 × 10^−3^ to 1.27 × 10^−3^ kg 1,4-DB eq. and FE from 5.59 × 10^−4^ to 5.66 × 10^−4^ kg 1,4-DB eq. These increases reflect the more intensive chemical handling and wastewater treatment requirements associated with KOH activation. Notably, ionizing radiation (IR) shows a more pronounced increase, rising from 6.43 × 10^−3^ kBq Co-60 eq. for NaOH to 8.48 × 10^−3^ kBq Co-60 eq. for KOH, indicating greater electricity-related upstream impacts.

Ecosystem-related indicators follow the same overall pattern. Marine ecotoxicity (ME) increases from 2.02 × 10^−3^ to 2.04 × 10^−3^ kg 1,4-DB eq., terrestrial acidification (TA) from 2.07 × 10^−3^ to 2.11 × 10^−3^ kg SO_2_ eq., and smog formation (SA) from 8.47 × 10^−2^ to 8.62 × 10^−2^ kg O_3_ eq. for NaOH and KOH routes, respectively. Metal depletion (MD) exhibits a comparatively larger relative increase, rising from 2.62 × 10^−3^ kg Cu eq. for NaOH to 3.51 × 10^−3^ kg Cu eq. for KOH, highlighting the higher mineral resource intensity of KOH production. The results demonstrate that NaOH activation is consistently environmentally preferable at the production stage, with lower energy demand and reduced impacts across most midpoint categories. However, these mass-based results do not account for adsorption performance, underscoring the importance of complementary performance-based assessment to fully evaluate the environmental efficiency of the two activation routes.

**Fig. 3:**
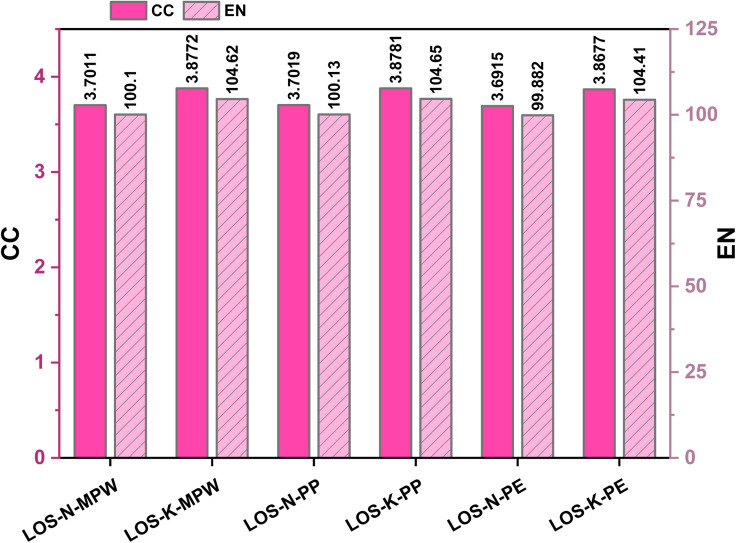
Comparison of CC and EN for OS–MPW composite and varying plastic waste including MPW, PP, PE using KOH and NaOH activation.

Fig. 3 examines whether the plastic type (MPW, PP, or PE) affects the CC and EN of the OS–plastic composites. The results show only minor differences across plastic types. For the NaOH route, CC remains 3.69–3.70 kg CO_2_ eq. and EN 99.9–100 MJ, while for the KOH route CC remains 3.87–3.88 kg CO_2_ eq. and EN 104–105 MJ. Overall, changing MPW to PP or PE does not significantly change CC or EN, whereas the activation chemistry (KOH *vs.* NaOH) remains the main factor controlling the environmental burden.

**Fig. 4:**
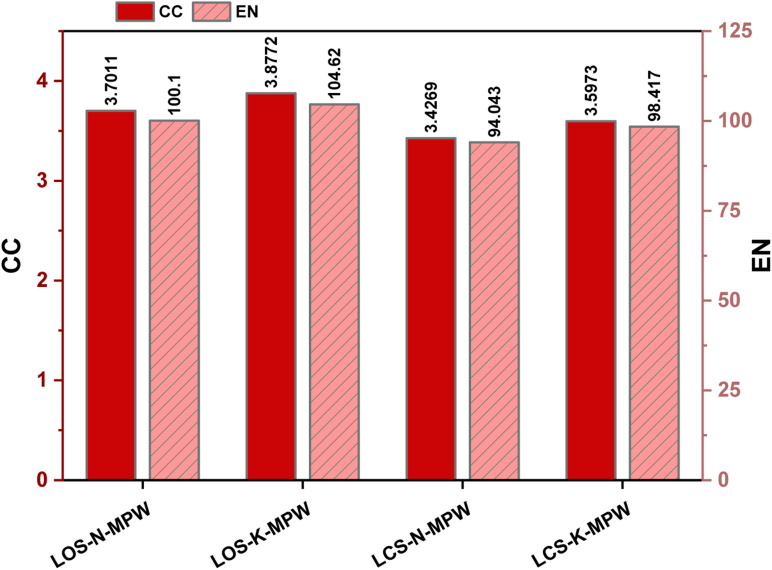
Comparison of climate change (CC) and net energy (EN) emissions for carbon–polymer composites prepared from mixed plastic waste (MPW) using olive stones (OS) and coconut shells (CS) under NaOH and KOH activation.

To further contextualize the environmental performance of the OS–MPW composite, a benchmarking comparison with coconut shell (CS) biomass was conducted under identical processing conditions. As shown in Fig. 4, the CS-based composites exhibit CC values of 3.42 and 3.59 kg CO_2_ eq. and EN values of 94 and 98.4 MJ for NaOH and KOH activation, respectively. The slightly lower impacts observed for CS are mainly attributed to differences in carbon yield and associated pyrolysis energy demand. Nevertheless, the overall magnitude of CC and EN remains comparable, indicating that biomass selection introduces moderate variation while the dominant influence continues to arise from activation chemistry and thermal processing.

**Fig. 5:**
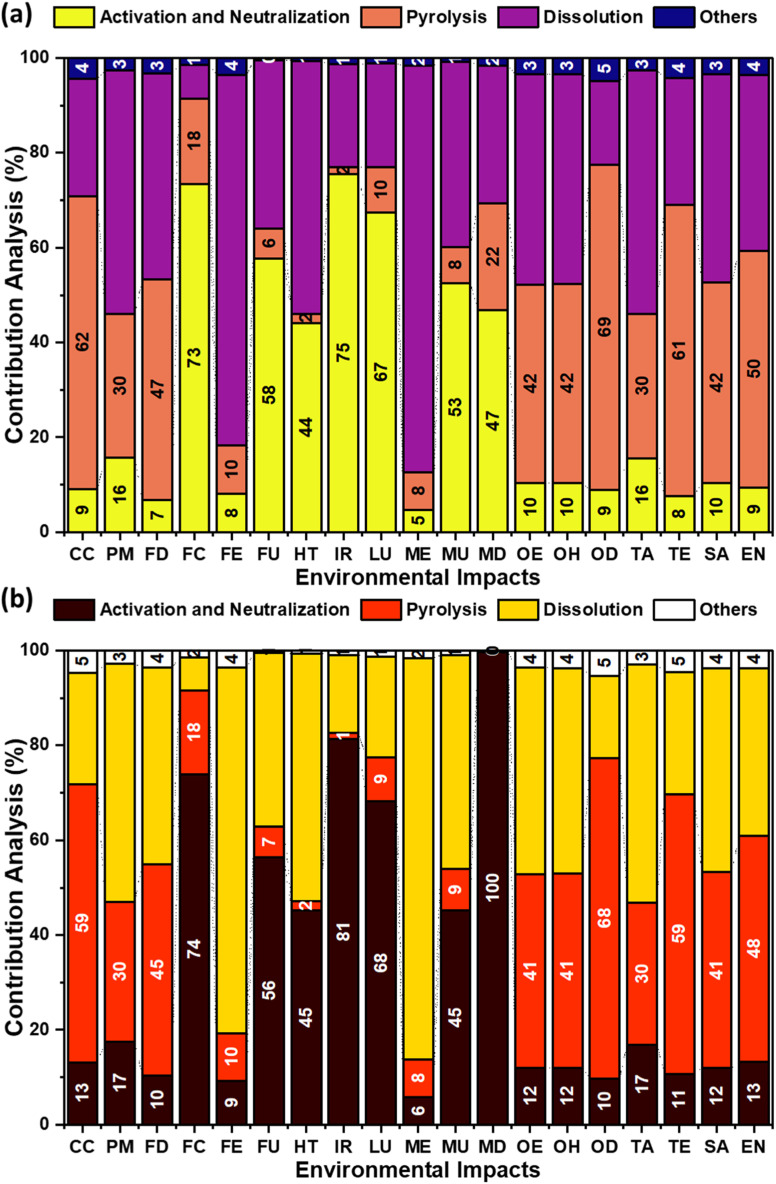
Contribution analysis of different steps involved in the preparation of AC composite: (a) using NaOH; (b) using KOH.

Fig. 5 illustrates the contribution analysis of individual process steps involved in producing OS–MPW AC composites using NaOH (Fig. 5a) and KOH (Fig. 5b) activation routes. For the NaOH route, pyrolysis emerges as the dominant contributor to most impact categories, particularly CC, where it accounts for 62% of total impact, followed by dissolution (25%), activation and neutralization (9%), and other auxiliary processes (4%). A similar dominance of pyrolysis is observed for net energy demand (EN), contributing 50%, while dissolution contributes 37%, indicating that thermal energy input and solvent-related operations are the principal energy drivers in the NaOH-based system. The term ‘Others’ include transport, drying, shredding, and heat treatment. These results highlight that, despite chemical usage during activation, the environmental burdens of the NaOH route are largely governed by energy-intensive thermal and dissolution steps rather than chemical activation itself.

For the KOH route (Fig. 5b), a comparable but more pronounced trend is observed. In this case, activation and neutralization contribute substantially to several categories, reflecting the higher embodied energy and resource intensity of KOH. For example, in fossil depletion (FD), activation and neutralization account for 10%, while pyrolysis contributes 45%, dissolution 41%, and others 4%. The increased contribution of dissolution and pyrolysis relative to NaOH suggests a compounded effect of higher energy requirements and solvent use when KOH is employed. Additionally, the activation step shows elevated contributions across toxicity and resource-related categories (*e.g.*, freshwater and human toxicity, metal depletion).

A direct comparison of Fig. 5(a) and (b) reveals systematic differences between the two activation routes. While pyrolysis remains the dominant hotspot in both systems, its relative contribution to CC is higher in the NaOH route (62%) compared to KOH, whereas the combined contribution of activation and dissolution is higher for KOH, particularly in FD and EN-related categories. Dissolution contributes up to 41% of FD in the KOH route, compared to lower shares in the NaOH system, indicating greater dependence on solvent-related energy and material inputs. Overall, the NaOH route exhibits a more balanced distribution of impacts, whereas the KOH route shows greater sensitivity to chemical activation and dissolution steps. These findings explain the higher mass-based environmental burdens observed for KOH activation and reinforce the importance of targeting pyrolysis energy efficiency and solvent management as key leverage points for reducing EIs in both systems.

**Fig. 6:**
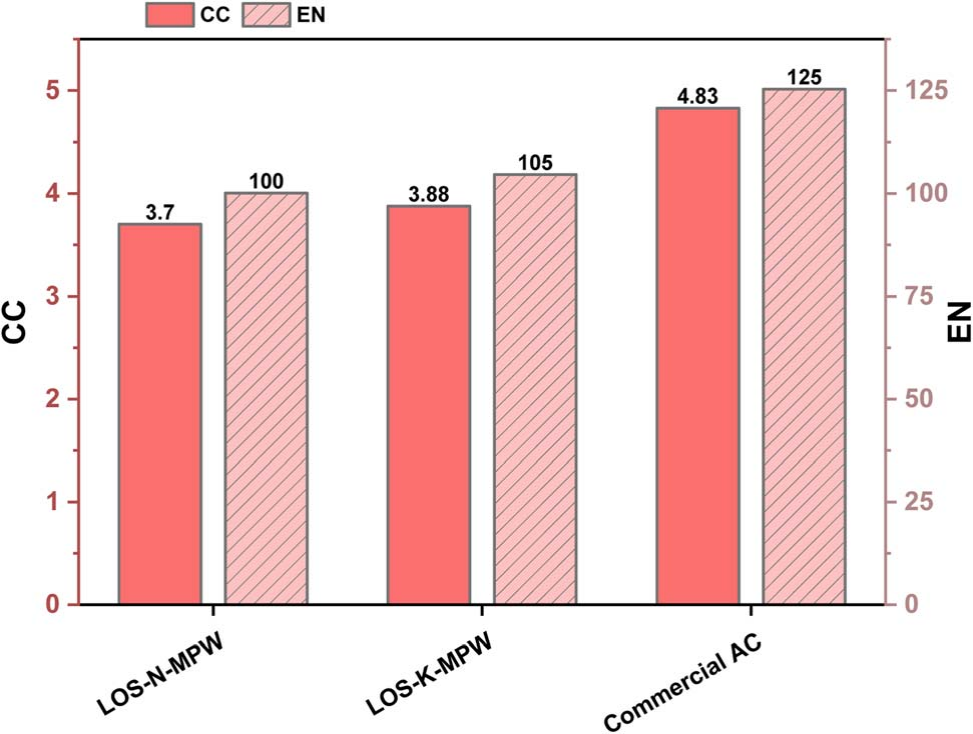
Comparison of as-prepared AC composites with commercial AC.

Fig. 6 compares the CC impact and EN of the as-prepared OS–MPW composites produced *via* NaOH and KOH activation, benchmarked against commercial AC. Both waste-derived composites exhibit lower CC and EN values than commercial AC, highlighting the environmental benefits of valorizing agricultural and plastic waste streams. The NaOH-activated composite shows a CC impact of approximately 3.7 kg CO_2_ eq. and an EN of about 100 MJ, while the KOH-activated composite exhibits slightly higher values of approximately 3.9 kg CO_2_ eq. and 105 MJ, respectively. In comparison, commercial AC exhibits substantially higher environmental burdens, with CC impacts of approximately 4.8 kg CO_2_ eq. and EN of about 125 MJ.

The elevated EIs associated with commercial AC are largely attributable to its conventional production route and feedstock selection. Commercial AC is commonly produced from fossil-based and energy-intensive precursors, such as hard coal, brown coal, and wood, as well as biomass sources including coconut or walnut shells. Its manufacturing typically involves carbonization under oxygen-free conditions, followed by high-temperature steam activation, both of which require substantial thermal energy input.^44,45^ In contrast, the OS–MPW composites benefit from the use of waste-derived raw materials, which avoid upstream extraction burdens and reduce reliance on virgin fossil resources.

**Fig. 7:**
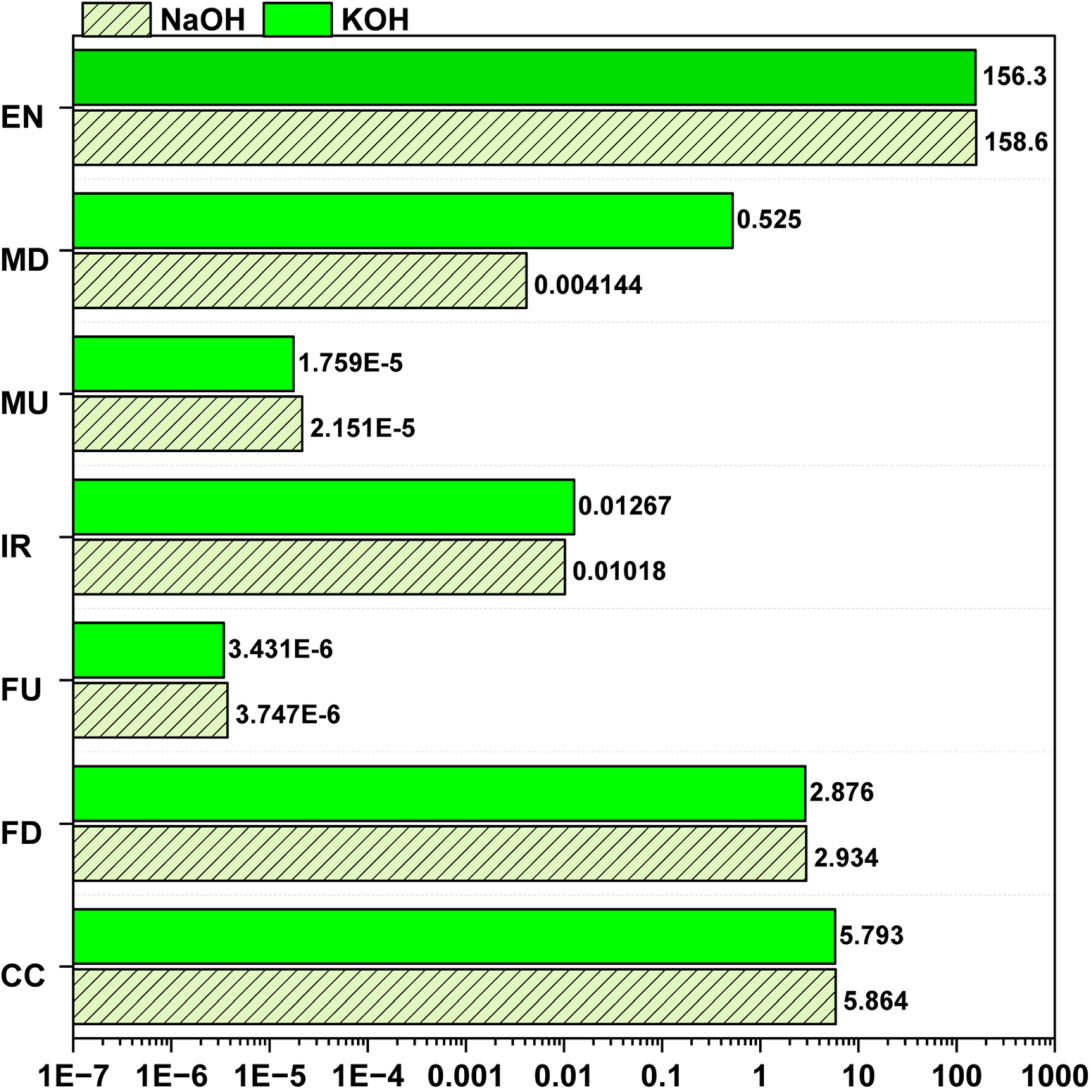
EIs *vs.* emissions on performance basis for 1 kg adsorption of Rhodamine 6G.

Fig. 7 presents the EIs of OS-derived AC composites on a performance basis, normalized to the adsorption of 1 kg of Rhodamine-6G, thereby explicitly accounting for differences in adsorption capacity between NaOH- and KOH-activated materials. When evaluated using this functional metric, the KOH-activated composite exhibits lower CC and EN impacts than the NaOH-activated counterpart. Specifically, CC decreases from 5.864 kg CO_2_ eq. for NaOH to 5.793 kg CO_2_ eq. for KOH, while EN decreases from 158.6 MJ to 156.3 MJ, reflecting the higher adsorption efficiency of KOH-AC and the consequently lower adsorbent requirement per unit dye removed. Table 3 summarizes the adsorption capacities of the AC composites. The KOH-activated composite exhibits a higher adsorption capacity for Rhodamine 6G (669 mg g^−1^) compared to the NaOH-activated composite (631 mg g^−1^). The high adsorption capacities observed are primarily attributed to the well-developed porosity and favorable surface chemistry of the OS–MPW composites. In conventional granular or pelletized ACs, the use of inert binders and the larger particle size often reduce the accessible surface area and the number of active adsorption sites, resulting in lower adsorption capacities compared with powdered AC. In the present composite system, the dissolution of polyolefin phases enables homogeneous dispersion of activated carbon and prevents pore blockage typically associated with binder-based shaping. Upon solidification, this process generates an open and interconnected carbon framework that preserves accessible porosity while providing mechanical integrity and ease of recovery. Consequently, the flake-like AC composites combine adsorption efficiencies comparable to powdered AC with the practical advantages of structured adsorbents. Rhodamine 6G was selected as a representative organic dye because it is widely used in laboratory, textile, and sensing applications and is frequently reported in adsorption studies, enabling comparison with existing literature. It possesses a high molar extinction coefficient, which allows reliable detection even at low concentrations, ensuring accurate mass-balance measurements. Moreover, dyes of similar aromatic structure are common contaminants in industrial effluents and surface waters, making Rhodamine 6G a suitable proxy for evaluating adsorption performance in water-treatment-related studies.^46,47^

**Table 3:** Adsorption capacity comparison

Rhodamine 6G	Adsorption (mg g^−1^)
K-MPW	669.3
N-MPW	631.2

Also, the regeneration performance of the OS–MPW composite was evaluated over three adsorption–desorption cycles (Table 4). The adsorption efficiency remained approximately 80% after the first cycle and gradually decreased to 65% and 44% after the second and third cycles, respectively. The decline in efficiency is attributed to partial pore blockage and surface fouling during repeated use. Nevertheless, the ability to regenerate the composite demonstrates potential for material reuse, which can contribute to lowering the overall environmental burden in practical applications.

**Table 4:** Regeneration efficiency of OS–MPW AC composite after adsorption–desorption cycles

Regeneration cycle	Adsorption efficiency (%)
1st	80
2nd	65
3rd	44

A similar trend is observed across several resource and nutrient-related impact categories. Fossil depletion (FD) decreases from 2.934 kg oil eq. for NaOH to 2.876 kg oil eq. for KOH, while freshwater eutrophication (FU) and marine eutrophication (MU) are reduced from 3.747 × 10^−6^ to 3.431 × 10^−6^ kg P eq. and from 2.151 × 10^−5^ to 1.759 × 10^−5^ kg N eq., respectively. These reductions indicate that, despite the higher mass-based impacts associated with KOH activation, its superior adsorption performance effectively offsets part of the upstream environmental burden when impacts are expressed per unit of pollutant removed. This highlights the importance of adopting performance-based functional units for adsorption-driven applications.

In contrast, metal depletion (MD) and ionizing radiation (IR) exhibit different behavior. On a performance basis, MD increases from 0.004144 kg Cu eq. for NaOH to 0.525 kg Cu eq. for KOH, while IR increases from 0.01018 to 0.01267 kBq Co-60 eq. This indicates that the higher adsorption capacity of KOH-AC is insufficient to compensate for the metal and electricity-intensive upstream production of KOH, which remains a dominant contributor to these categories.

**Fig. 8:**
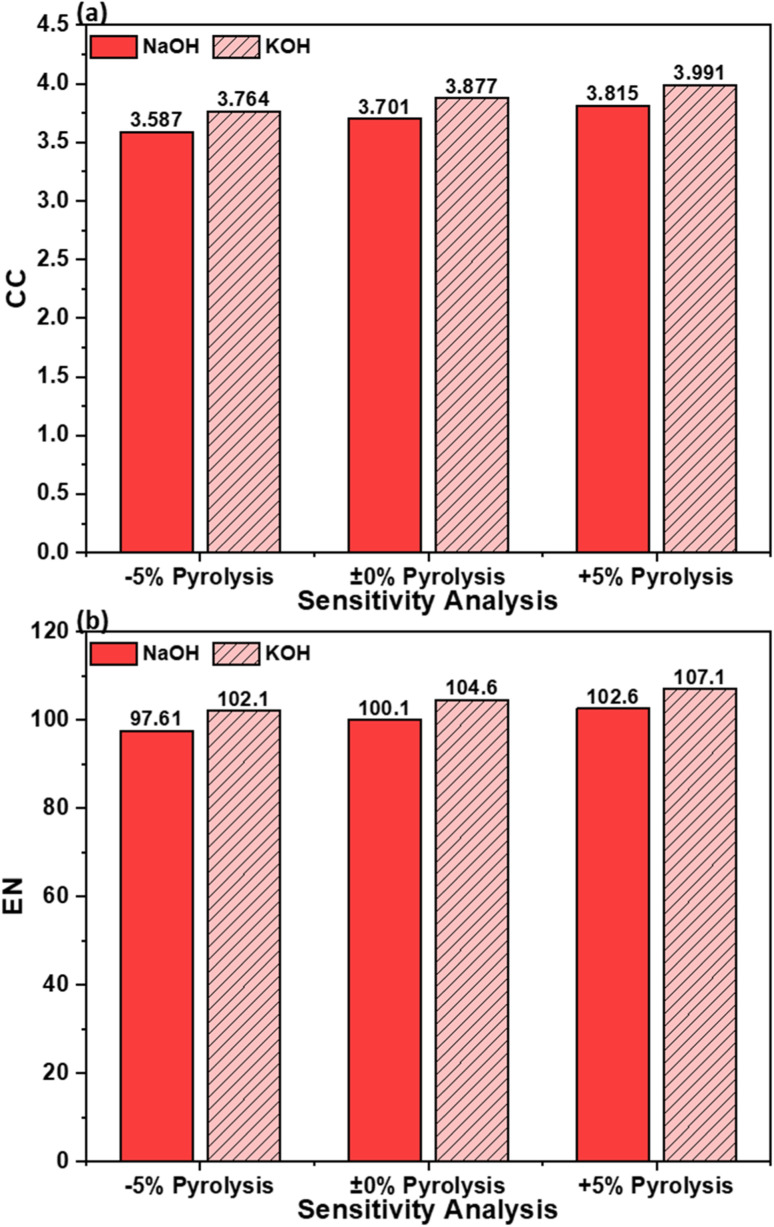
Sensitivity analysis of AC composite with 5% uncertainty in power consumption during pyrolysis step: (a) CC impact and (b) EN impact.

Fig. 8 presents the sensitivity of CC and EN to 5% variation for OS–MPW composites produced *via* NaOH and KOH activation. Pyrolysis was selected for sensitivity analysis because it constitutes the most energy-intensive step in AC production and is inherently prone to operational variability. Variations in feedstock heterogeneity, furnace performance, and heat losses during scale-up and routine operation can all influence electricity demand during pyrolysis. To capture this realistic uncertainty, a 5% variation in pyrolysis power consumption was applied, representing plausible fluctuations in energy use rather than an artificial or fixed process deviation. This approach enables evaluation of the robustness of the LCA results with respect to uncertainty in the dominant energy input.

At the baseline condition (0% pyrolysis), the KOH route exhibits CC and EN values of 3.877 kg CO_2_ eq. and 104.6 MJ, respectively, compared to 3.701 kg CO_2_ eq. and 100.1 MJ for the NaOH route. These baseline differences are consistent with earlier mass-based results and reflect the slightly higher energy intensity associated with KOH activation. A reduction of 5% in pyrolysis electricity consumption leads to proportional decreases in both indicators. For the KOH route, CC decreases from 3.877 to 3.764 kg CO_2_ eq., while EN decreases from 104.6 to 102.1 MJ. Similarly, NaOH-activated composites show reductions in CC from 3.701 to 3.587 kg CO_2_ eq. and in EN from 100.1 to 97.61 MJ. Conversely, a 5% increase in pyrolysis energy demand results in corresponding increases, with KOH CC and EN rising to 3.991 kg CO_2_ eq. and 107.1 MJ, and NaOH increasing to 3.815 kg CO_2_ eq. and 102.6 MJ, respectively. The near-linear response of CC and EN to changes in pyrolysis energy confirms the dominant role of thermal energy input in shaping these impacts.

**Fig. 9:**
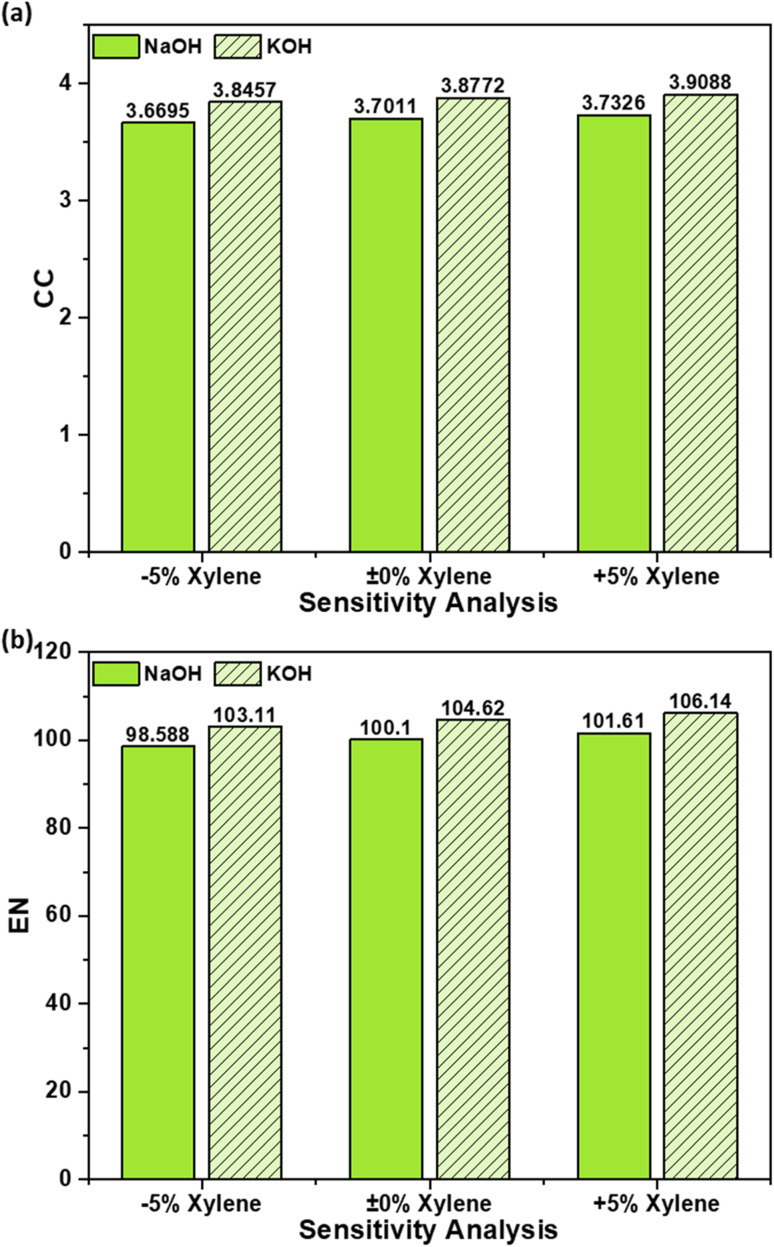
Sensitivity analysis of AC composite with 5% uncertainty in xylene consumption in dissolution step: (a) CC impact and (b) EN impact.

Fig. 9 presents the sensitivity of CC impact and EN to 5% variation in xylene consumption during the dissolution step for OS–MPW carbon–polymer composites produced *via* NaOH and KOH activation. Xylene was selected for sensitivity analysis because it plays a central role in the dissolution and dispersion of AC within the polymer matrix, directly influencing both material integration and process efficiency. As an aromatic, fossil-derived solvent, xylene contributes not only to fossil depletion and CC impacts, but also to process energy demand through solvent heating, recovery, and losses during handling. In practical operation, xylene consumption is subject to variability arising from solvent recovery efficiency, evaporation losses, equipment design, and scale-dependent processing conditions, making it a relevant parameter for uncertainty analysis. A 5% variation in xylene usage was therefore applied to represent realistic operational fluctuations rather than fixed or idealized solvent consumption.

At the baseline condition (0% xylene), CC values are 3.7011 kg CO_2_ eq. for NaOH and 3.8772 kg CO_2_ eq. for KOH, while EN values are 100.1 MJ and 104.62 MJ, respectively. A 5% reduction in xylene consumption leads to modest but consistent decreases in both indicators. For NaOH activation, CC decreases from 3.7011 to 3.6695 kg CO_2_ eq. and EN from 100.1 to 98.588 MJ, while the KOH route shows reductions in CC from 3.8772 to 3.8457 kg CO_2_ eq. and EN from 104.62 to 103.11 MJ.

Conversely, increasing xylene use by 5% raises CC to 3.7326 kg CO_2_ eq. (NaOH) and 3.9088 kg CO_2_ eq. (KOH), with EN increasing to 101.61 MJ and 106.14 MJ, respectively. The nearly linear response of CC and EN to solvent variation indicates that xylene acts as a secondary but non-negligible contributor to EIs. Compared to the sensitivity observed for pyrolysis energy demand, the absolute variations associated with xylene (0.06 kg CO_2_ eq. and 3 MJ across the full range) are smaller, yet still relevant for fine-tuning environmental performance. Overall, these results emphasize that while pyrolysis remains the dominant hotspot, improved solvent recovery and minimization strategies can provide additional, incremental reductions in the environmental footprint of carbon–polymer composite production.

The results highlight an important trade-off between climate-related and toxicity-related environmental indicators. Activation routes and composite formulations that exhibit lower CC and EN do not necessarily minimize impacts across all categories. In particular, toxicity- and resource-related indicators such as human toxicity, freshwater ecotoxicity, ionizing radiation, and metal depletion are more strongly influenced by upstream chemical production and electricity supply chains rather than direct process energy use. As a result, KOH-based systems, while exhibiting slightly higher CC and EN on a mass basis, show disproportionately higher impacts in metal depletion and ionizing radiation due to the resource- and energy-intensive production of potassium-based chemicals. These findings emphasize that improving climate performance alone is insufficient to guarantee overall environmental sustainability and that multi-indicator assessment is essential for identifying balanced and application-appropriate optimization pathways.

## 4. Conclusion

This study presents a comprehensive LCA of AC–polymer composites using NaOH and KOH chemical activation routes, addressing a critical gap in the environmental evaluation of hybrid waste-derived adsorbents. Environmental performance was assessed using both mass-based (per kg of composite produced) and performance-based (per kg of dye adsorbed) functional metrics, enabling a comparison that integrates material production efficiency with adsorption functionality.

On a mass-based basis, NaOH activation consistently exhibited lower EIs than KOH across most midpoint categories. CC and EN were lower for NaOH-activated composites, reflecting reduced embodied energy and resource intensity associated with NaOH production and downstream processing. Contribution analysis identified pyrolysis and dissolution as the dominant environmental hotspots, while activation and neutralization played a secondary but non-negligible role, particularly for KOH. Benchmarking against commercial AC further demonstrated that OS–MPW composites offer substantial reductions in CC and EN, highlighting the environmental benefits of circular valorization of agricultural and plastic waste streams.

When impacts were normalized to adsorption performance, a partial reversal in environmental preference was observed. Due to its higher adsorption capacity, KOH-AC exhibited lower CC, EN, fossil depletion, and eutrophication impacts per kg of dye adsorbed, despite its higher mass-based burdens. However, this performance advantage did not extend to all categories. Metal depletion and ionizing radiation remained substantially higher for KOH, indicating that performance normalization cannot fully offset the upstream environmental intensity associated with KOH production.

Sensitivity analyses further reinforced the robustness of the conclusions. Variations of 5% in pyrolysis energy consumption produced changes in CC and EN comparable to the differences between activation routes, confirming pyrolysis as the most influential energy hotspot. In contrast, xylene (solvent) consumption exhibited a smaller but systematic effect, identifying solvent management as a secondary optimization pathway.

## Author contributions

Junaid Saleem: conceptualization, investigation, formal analysis, supervisor, writing – review & editing. Zubair Khalid Baig Moghal: formal analysis, investigation, validation, writing – review & editing. Furqan Tahir: formal analysis, investigation. Gordon McKay: validation, formal analysis.

## Conflicts of interest

There are no conflicts to declare.

## Data Availability

The original contributions presented in the study are included in the article, further inquiries can be directed to the corresponding author.
